# Intraoperative analysis of patellofemoral joint morphology before and after total knee arthroplasty: A feasibility study

**DOI:** 10.1007/s00132-022-04224-x

**Published:** 2022-03-07

**Authors:** Maeruan Kebbach, Enrico Mick, Stephan Kirschner, Joerg Luetzner, Rainer Bader

**Affiliations:** 1 Biomechanics and Implant Technology Research Laboratory, Department of Orthopaedics, Rostock University Medical Center, Doberaner Straße 142, 18057 Rostock, Germany; 2 Orthopaedic Clinic, St.Vincentius-Kliniken, Steinhäuser Straße 18, 76135 Karlsruhe, Germany; 3 University Center of Orthopaedics and Traumatology, University Medicine Carl Gustav Carus Dresden, TU Dresden, Fetscherstraße 74, 01307 Dresden, Germany

**Keywords:** Patellofemoral joint, Patellofemoral morphology, Femoral trochlear groove, Total knee replacement, Digital tactile scanning, Patellofemoralgelenk, Patellofemorale Morphologie, Femorale Trochlearinne, Knieendoprothetik, Digitale Abtastmethode

## Abstract

**Background:**

Patellofemoral problems after total knee arthroplast (TKA) are frequent and often associated with a change in the geometry of the trochlear groove.

**Objective:**

The present study aimed to analyze the feasibility of intraoperative examination of the patellofemoral joint geometry before and after the implantation of bicondylar total knee replacements without exposing the patient to radiation.

**Material and methods:**

The patellofemoral joint morphology geometries of 33 patients before and after implantation of a bicondylar total knee replacement was intraoperatively analyzed using a digital scanning method. Femoral surface geometries were extracted from intraoperatively recorded tactile scanning data using an in-house algorithm. The geometries were then characterized by sulcus height, medial femoral condyle height and lateral femoral condyle height.

**Results:**

Our preliminary results show that these key geometric parameters are only partially restored after TKA leading to a distinctly different shaped profile of the anterior distal femur. Maximum and minimum mean differences in sulcus height, medial femoral condyle height, and lateral femoral condyle height before and after surgery were 2.00 mm/−3.06 mm, 2.51 mm/−6.25 mm and 2.74 mm/−3.58 mm, respectively.

**Conclusion:**

A new method for intraoperative analysis of the patellofemoral joint morphology before and after TKA without radiation exposure was developed and utilized. Even with the use of modern total knee designs, the patellofemoral articulation is considerably changed postoperatively as quantified by the key parameters of sulcus height, medial femoral condyle height, and lateral femoral condyle height. This may result in altered knee biomechanics and might explain persistent patellofemoral disorders, which are often reported by patients after TKA.

## Abstract (de)

**Hintergrund:**

Probleme des Patellofemoralgelenks nach der Implantation einer Knietotalendoprothese (Knie-TEP) sind häufig und gehen mit geometrischen Veränderungen der Trochlea einher.

**Ziel:**

Das Ziel der vorliegenden Pilotstudie war es, die Machbarkeit einer intraoperativen Untersuchung der Geometrie des Patellofemoralgelenks (PFG) vor und nach der Implantation von bikondylären Knietotalendoprothesen ohne zusätzliche Strahlenexposition des Patienten zu analysieren.

**Material und Methoden:**

Dazu wurde intraoperativ eine digitale Abtastmethode verwendet, mit der die Morphologie des PFG von 33 Patienten vor und nach der Implantation einer Knie-TEP analysiert wurde. Die Geometrien der Femuroberfläche wurden aus den intraoperativ aufgezeichneten Abtastdaten mit Hilfe eines Algorithmus extrahiert und im Hinblick auf die Sulkushöhe sowie die mediale und laterale Höhe der Femurkondyle charakterisiert.

**Ergebnisse:**

Die vorläufigen Daten zeigen, dass diese geometrischen Schlüsselparameter nach der Implantation einer Knie-TEP nur teilweise wiederhergestellt werden, was zu einem deutlich veränderten Oberflächenprofil des distalen Femurs führt. Die maximale und minimale mittlere Abweichung in der Sulkushöhe, der medialen und der lateralen Femurkondylenhöhe vor und nach der Knie-TEP betrugen 2,00/−3,06 mm; 2,51/−6,25 mm bzw. 2,74/−3,58 mm.

**Diskussion:**

Es wurde eine neue Methode zur intraoperativen Analyse der Morphologie des PFG vor und nach der Implantation einer Knie-TEP ohne zusätzliche Strahlenbelastung entwickelt. Bei der Verwendung moderner Knie-TEPs ist postoperativ die patellofemorale Artikulation erheblich verändert, was durch die Schlüsselparameter Sulkushöhe, mediale und laterale Femurkondylenhöhe gezeigt werden konnte. Dies kann zu veränderten biomechanischen Randbedingungen am Kniegelenk führen und könnte persistierende patellofemorale Beschwerden erklären, über die Patienten nach einer Knie-TEP häufig berichten.

## Introduction

Reduced satisfaction after total knee arthroplasty (TKA) is often associated with dysfunction during daily activities that impairs the patient’s quality of life [23]. In the US market, the number of TKA procedures is expected to rise by 673% from 2005 to 2030 reflecting future need due to an ageing population. For the same period, the demand for revision surgery is estimated to grow by 601% [17]. Despite this expected increase in operations, patellofemoral joint disorders, e.g. patellar subluxation and dislocation, implant wear or anterior knee pain, remain present and constitute a major factor of insufficiency after TKA [10, 22, 28, 35, 37].

Tibiofemoral and patellofemoral kinematics have been extensively investigated and are known to be affected by implant design, implant positioning, and knee joint morphology [11, 21, 22, 25, 28, 34]. Some research groups investigated differences between preoperative and postoperative geometries on human cadaveric specimens or in computational studies [1, 4, 7, 9, 14, 29, 30, 34]; however, as suggested in [10, 21, 22, 25], TKA does not restore native joint geometry, and only preoperative and postoperative comparisons can reliably identify joint morphology. Since patellofemoral joint geometry and implant component positioning alter knee joint biomechanics, it is difficult to identify the extent to which key geometric parameters are affected.

In this context, Saffarini et al. [26] investigated the influence of implant geometries on knee kinematics after comparing two different implant systems. They emphasized the impact of patellofemoral geometry on mid-flexion knee kinematics. In a computational study, Varadarajan et al. [34] investigated the trochlear geometry before and after TKA and they revealed that the trochlear anatomy was partially restored. Further biomechanical studies showed that total knee designs do not restore normal patellar tracking which might lead to anterior knee pain [1, 4, 7, 34]. Recently, Du et al. [9] investigated the discrepancy between the trochlear geometries of the native and prosthetic knee in a computational study with virtual TKA implantation for different implant designs. They found significant differences between native and endoprosthetic knees for different geometrical parameters of the trochlear groove, which alters knee joint mechanics. Moreover, standard TKA implant designs and instruments do not consider the position and orientation of the trochlear groove or the patellar height. The design of the trochlear geometry is an important parameter when restoring patellofemoral joint kinematics. It has been reported that trochlear geometry can prevent patella-associated complications [9, 34].

Restoration of the knee biomechanics is inherently bound to joint geometry [22, 29, 30, 36]. Previous approaches used computed tomography (CT) data to characterize the geometry of the trochlear groove [7, 13, 14, 19]; however, CT is not capable of registering cartilaginous tissue properly and is invasive for the patient [27, 32]. Therefore, virtual surgery [7, 9, 34] may not entirely represent the actual clinical application. Common approaches for data acquisition using medical imaging, e.g. via CT as used by Iranpour et al. [14], Chen et al. [7]. and Du et al. [9] suffer from risk due to radiation exposure, insufficient visualization of cartilaginous tissue, and high sensitivity to projection artefacts [36]. The use of magnetic resonance imaging (MRI), as used by Varadarajan et al. [34] for medical imaging can solve this problem; however, this is costly and time-consuming and, in the case of metallic implants, likely to fail due to artefacts. The use of cadaveric knees, although widely performed for experimental testing in TKA research, [12, 27] bears issues in terms of representativeness, since cadaver tissue is different from patient tissue [32, 36]. That is, patients scheduled for TKA have osteoarthritis associated with changes in the joint surfaces due to chondral and bone defects as well as osteophytes suggesting that studies on non-osteoarthritic knees might not properly reflect the reality [7, 9, 18, 34]. Consequently, alternative approaches for analyzing trochlear groove morphology are required.

Therefore, the present study aimed to analyze the feasibility of intraoperative examination of the patellofemoral joint morphology before and after TKA by sampling and reconstructing the femoral anterior cortex using tactile scanning. This approach should overcome the limitations of previous studies based on sole CT imaging, virtual surgery or cadaver examinations.

## Material and methods

The study presented was approved by the institutional review board (EK 182072008). A total of 33 patients (15 males and 18 females) were investigated while they underwent TKA due to osteoarthritis. Informed consent was obtained from all patients. The females were 69.8 ± 6.7 years old and had an average weight of 87.2 ± 13.2 kg; the males were 70.5 ± 9.7 years old and had an average weight of 93.2 ± 11.1 kg.

The cruciate-retaining (CR) bicondylar total knee replacement system Scorpio CR (Stryker, Mahwah, NJ, USA) was utilized in all cases. Surgical procedures were performed by two experienced orthopedic surgeons with the aid of a special navigation system (Precision Knee Navigation software v4.0, Stryker Orthopaedics). All bone resections were performed as guided by the navigation system. The femoral component was oriented perpendicular to the mechanical axis in the coronal and sagittal planes. The rotational alignment of the respective implant was set parallel to the transepicondylar axis (TEA). No patellar resurfacing was performed. Additional time needed for the navigation amounts to approximately 10 min.

### Verification of the tactile scanning procedure

**Fig. 1:**
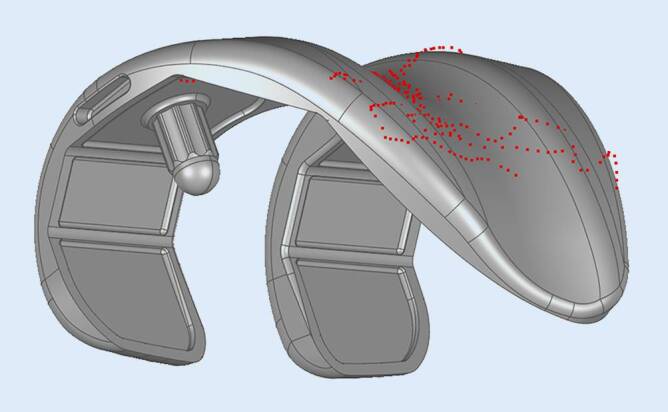
Verification of the tactile scanning procedure by aligning the recorded postoperative point cloud to the CAD data of the implant surface. Exemplarily, the point cloud obtained from patient case #029 (*red*) aligned to a corresponding Scorpio CR femoral surface component (size 7)

The accuracy of the scanning procedure was verified by fitting the sampled point clouds of the femoral implant surface to the computer-aided design (CAD) files of the corresponding femoral components. Implant CAD surfaces and recorded point clouds were spatially aligned using best-fit alignment optimization of the 3D software Geomagic® Studio 2013 (3D Systems, Rock Hill, SC, USA). Reconstruction accuracy was evaluated based on the relative distance of a recorded sample point to the true implant surface. To ensure the algorithm’s function, this procedure was repeatedly performed for 21 right knees. The mean minimum and maximum deviations from the postoperatively sampled points to the respective implant surface were 0.27 mm and 1.89 mm, respectively. Concerning all patient cases, a mean distance error of 0.79 mm was determined, while the average root mean square error (RMSE) was 0.65 mm. An example of a fitted point cloud (*red*) to the implant surface is illustrated in Fig. 1.

### Intraoperative procedure of tactile scanning measurement using a navigation system

The respective trochlear groove surface geometries were recorded using the mentioned navigation system with a tactile scanner. A bicortical pin inserted into the distal femur was used as coordinate origin and remained in its position during the entire surgical procedure. Relative to this reference point, a set of anatomical landmarks (i.e. femoral head center, medial epicondyle point and lateral epicondyle point) were automatically registered prior to bone cuts to construct the reference system and to perform the measurements required. Before bone resection and after implantation of the femoral component, the anterior femoral cortex and the trochlear groove geometries were registered. The obtained data served as a reference for the subsequent surface reconstructions and determination of the parameter sets. Specifically, the geometry was characterized by its key parameters: sulcus height (SH), medial femoral condyle height (medCH), and lateral femoral condyle height (latCH).

**Fig. 2:**
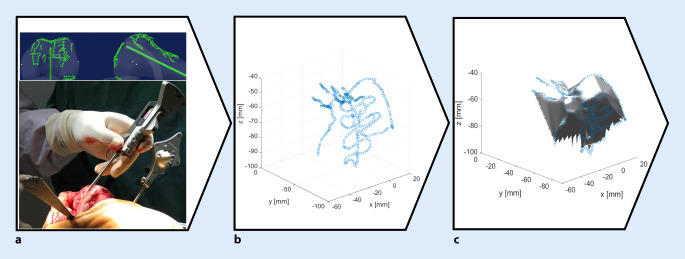
Workflow for the surface reconstruction of the femoral anterior cortex by means of the intraoperatively recorded point cloud. Using a commercially available navigation system (Precision Knee Navigation software v4.0, Stryker Orthopaedics, Mahwah, NJ, USA) with the tactile scanner (**a**), the anterior cortex was sampled with emphasis on the trochlear groove before and after total knee arthroplasty. The recorded point clouds (**b**) served as the basis for the surface reconstruction using an in-house code (**c**)

The relevant surfaces of the knee joint were reconstructed from point clouds, which were obtained by means of tactile surface scanning that was manually performed by the surgeons. During each intraoperative measuring procedure, approximately 400–500 points on the anterior cortex, including the patellofemoral contact area, were sampled and recorded (Fig. 2a, b) for offline processing and evaluation. In the following section, the data processing, surface reconstruction, and parameter determination procedures are described in detail.

### Data processing, surface reconstruction and parameter determination

The data were imported in MATLAB (The MathWorks® Inc., Natick, MA, USA) and processed according to the following workflow: using the tactile scanner, the surgeons registered points of important anatomical landmarks for the establishment of the reference system and manually sampled the anterior femoral cortex intraoperatively by using the pointing device (Fig. 2a). The sampled point data were imported in MATLAB as a text file from which 3D location coordinates were retrieved to reconstruct the surface of the anterior cortex by triangulation from the intraoperatively sampled points. The space between the points was represented by linear interpolation. The surface was therefore generated as a polygon model where the triangles were represented by the nodes as their corners (Fig. 2b, c).

**Fig. 3:**
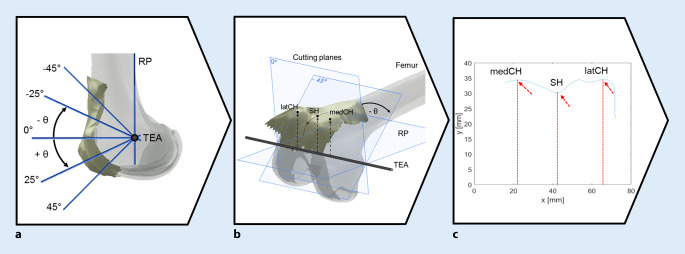
Process of the automated evaluation of geometric trochlear groove parameters. Starting from the reference plane (RP) referring to θ = −45° cranial, the cutting plane rotated around the transepicondylar axis (TEA) to θ = +45° caudal in 1° increments (**a**). The key parameters sulcus height (SH), medial condyle height (medCH) and lateral condyle height (latCH) were extracted from the computed cutting contour using a custom MATLAB program (**b**). The key parameters were determined for each angular increment of the cutting plane and the native as well as the artificial anterior cortex geometry (**c**)

By extending our MATLAB code, the geometric parameter sets for the evaluation of SH, medCH, and latCH could be automatically extracted (Fig. 3). To do so, a reference plane containing the lateral and medial epicondylar points, the femoral head center, and the lateral and medial epicondyles defining the TEA was established. Then, a second plane, the so-called cutting plane was defined as being perpendicular to the reference plane and comprising the TEA. Accordingly, any rotational displacement between this cutting plane and the reference plane produces a cutting angle (θ), where θ = 0° corresponds to the state where the cutting plane and reference plane are perpendicular (Fig. 3a, b). Cutting planes were generated incrementally in 1° steps for angles ranging from θ = −45° (cranial) to θ = +45° (caudal). Each of the resulting cross-sections contained the contour of the reconstructed femoral anterior cortex, which was analyzed in terms of the parameters of interest: SH, medCH, and latCH (Fig. 3c).

The cutting contour was represented by points that were interpolated to a continuous curve. Smoothing of the surface, search for local extrema, and the deepest point of the trochlear sulcus, the SH, as well as the medCH and latCH of this surface and the respective cutting contours were performed. All values were evaluated about the established Cartesian system defined from the anatomic landmarks (Fig. 3b). For each cross-section, the geometric parameters were determined automatically using the previously described MATLAB code. Figure 3 shows a schematic description of the evaluation of the trochlear groove parameters.

Both preoperative and postoperative data were evaluated using the developed workflow (Fig. 2 and 3), therefore enabling a comparison of trochlear groove geometry before and after TKA. To ease the evaluation of results, all patients were summarized in groups. Furthermore, the intraindividual difference of preoperative and postoperative sulcus height (∆SH) as well as medial/lateral femoral condyle height (∆medCH and ∆latCH) were analyzed. The ∆SH, ∆medCH, and ∆latCH were calculated by subtracting the preoperative values from the postoperative values for each cutting angle. Mean differences and standard deviations were then calculated by averaging ∆SH, ∆medCH, and ∆latCH for each cutting plane. In this manner, relative changes in the investigated parameters were determined while the influence of anatomical deviation from patient to patient could be eliminated.

## Results

All geometric parameters showed differences along the trochlear groove surface before and after TKA. To evaluate the sole influence of TKA rather than individual geometry on the investigated parameters, the results are additionally presented in terms of the mean difference of preoperative and postoperative parameters (∆SH, ∆medCH, and ∆latCH). The parameters are defined as a function of the cutting angle θ, which corresponds to the location on the frontal femoral cortex (Fig. 3a). Due to the varying quality of raw data and the number of measured points per surface, the result evaluation was restricted to an interval, i.e., the cutting angle ranging from θ = −45° to θ = +15°. For the sake of clarity, standard deviations are displayed only in the positive direction

### Sulcus height

**Fig. 4:**
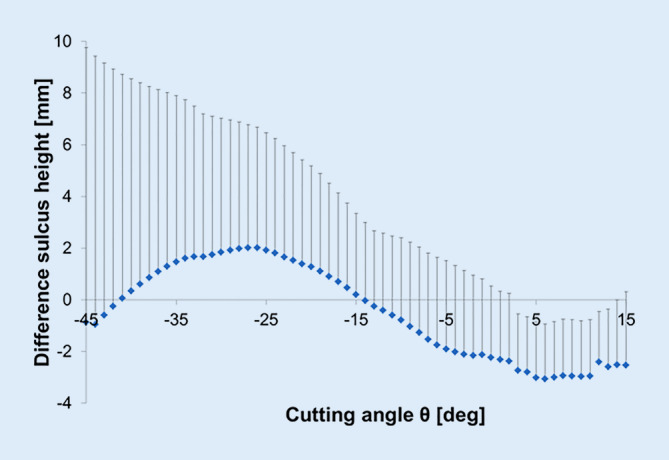
Graph showing the mean difference between preoperative and postoperative situation in sulcus height (∆SH) as a function of the cutting angle for implant system Scorpio CR

The difference in sulcus height ∆SH is depicted in Fig. 4. The diagram shows a pronounced change in SH after TKA. In the proximal portion of the trochlear groove, the postoperative SH was higher than in the natural knee. Contrarily, in the distal portion, the opposite behavior of the SH was observed. The maximum ∆SH = 2.00 mm was detected at a cutting angle of θ = −26°.

**Fig. 5:**
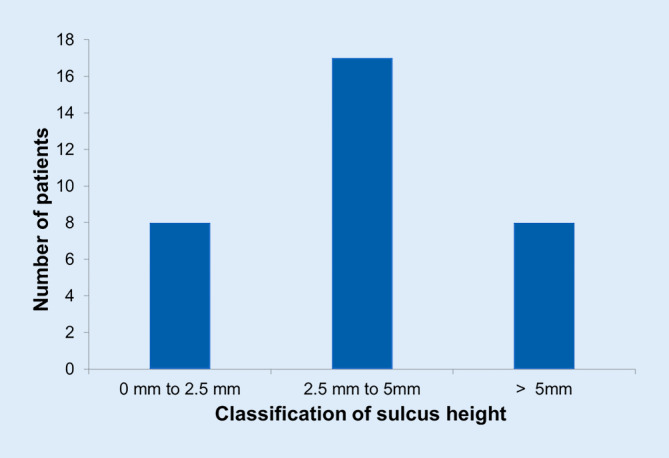
Classification into patient groups according to their relative change in sulcus height (preoperatively vs. postoperatively): high change (> 5 mm), medium change (> 2.5–5 mm) and small change (0–2.5 mm)

The maximum and minimum mean difference in ∆SH was changed by 2.00 mm and −3.06 mm after surgery, respectively. Generally, high standard deviations were observed that were distinctly higher in the proximal femoral cortex. For further analysis, patients were classified according to their RMSE in ∆SH (preoperatively vs. postoperatively) following the definitions: high change (> 5 mm), medium change (2.5–5 mm), and small change (0–2.5 mm) (Fig. 5).

The classification shows that 52% of the subjects had a medium change (2.5–5 mm) and 24% had a small change in SH after TKA.

### Medial and lateral femoral condyle height

**Fig. 6:**
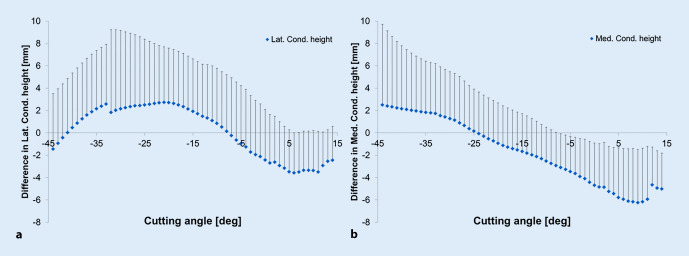
The mean difference in femoral condyle height along the trochlear groove between preoperative and postoperative situations. The mean difference in lateral (**a**) condyle height (∆latCH) and medial (**b**) condyle height (∆latCH) as a function of the cutting angle for implant system Scorpio CR

For the medCH and latCH, comparable differences were observed preoperatively and postoperatively. The condyles, in particular the medial condyle, were higher in the native knee compared to the prosthetic knees. Figure 6 shows ∆medCH and ∆latCH as a function of the cutting angle.

A difference in condyle height (i.e. ∆medCH or ∆latCH) after TKA is recognizable; however, as the change in the condyle heights takes a different shape over the cutting angle, the condyle profiles alongside the cutting angle differed. The femoral condyle height was generally increased in the cutting angles referring to extension. For lower cutting angles (i.e. −5° for the latCH and −25° for the medCH), the femoral condyle height decreased compared to the native knee. The maximum decrease of the condyle height varied between the condyle sides and was far more distinct in cutting angles referring to higher knee flexion. Additionally, the lateral condyle tended to be more increased, while the medial condyle was more decreased. The maximum/minimum mean differences in ∆medCH and ∆latCH differed with 2.51 mm/−6.25 mm and 2.74 mm/−3.58 mm after TKA, respectively. Similar to the SH, very high standard deviations were observed that were slightly higher in the proximal femoral cortex.

**Fig. 7:**
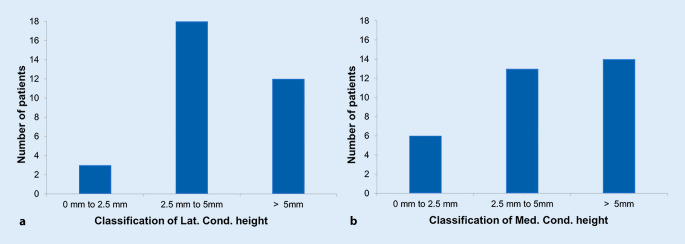
Classification into patient groups according to their relative change lateral/medial condyle height pre- vs. post-operatively: High change (> 5 mm), medium change (> 2.5–5 mm) and small change (0–2.5 mm). **a** Results for parameter lateral condyle height, **b** results for parameter medial condyle height

Following the previous results, patients were classified according to their RMSE (pre-op compared to post-op) of ∆latCH and ∆medCH following the definitions: high change (> 5 mm), medium change (2.5–5 mm), and small change (0–2.5 mm), see Fig. 7.

Most of the patients had a medium change in terms of the latCH with 54% of the subjects, and 9% had a small change after TKA. Regarding the medCH, 40% of the subjects had a medium change and 18% had a small change after TKA.

## Discussion

Patellofemoral disorders after TKA are frequent and might be associated with the change in joint articulation morphology. Herein, trochlear groove geometries of 33 patients were examined intraoperatively via tactile scanning before and after implantation of a bicondylar total knee replacement without radiation exposure to characterize the geometric key parameters SH as well as medCH and latCH. The main finding was that despite the use of a modern bicondylar total knee endoprosthesis, the artificial joint articulation morphology has a differently shaped contour. Changes in patellofemoral morphology may alter knee joint dynamics, e.g., a thicker anterior shield leads to increased patellofemoral contact force.

This feasibility study showed distinct alterations in the investigated parameters SH, medCH, and latCH due to TKA throughout the femoral anterior cortex. Concerning the SH, the total knee replacement caused an increase in the proximal portion of the trochlea and a decrease in the distal portion. Broadly similar and independent from implant size, the positive peak in ∆SH occurred between −30° and −15°, which was then followed by the negative peak at around +15°. This suggests that the total knee replacement implanted does not match the geometric contour of the natural trochlear groove. The results also showed that most patients (52%) had a medium change in SH due to TKA, which could be a potential contributor to anterior knee pain as previous studies stated [2, 16] that the anterior-posterior positioning of the femoral component is an important factor. Note, although it has been shown that there is a significant difference in knee joint kinematics between male and female [5, 8, 20], the calculated parameters of the trochlear groove showed similar results among genders as equally reported by several studies [7, 24, 34]. Therefore, distinction between male and female subjects was not done.

Varadarajan et al. [34] reported that the SH in knees after TKA was smaller than in natural knees almost over the whole range of their defined cutting planes; however, they investigated different implant designs, in which the SH may vary, and their study was solely simulative, which might lead to different results when compared to real TKA implantations. Du et al. [9] instead reported similar results to our study. Likewise, they observed that SH in natural knees is smaller than in endoprosthetic knees. The implications of altered SH are severe as they may affect knee flexion moment and patellofemoral joint forces [9]. A higher SH due to TKA leads to a higher retropatellar pressure as the findings of Steinbrueck et al. [29] showed, thus emphasizing the importance of this parameter. The absolute magnitudes of ∆SH, however, are not distinguishable due to high standard deviations within each group. We explain these high standard deviations with the variation of the quality and number of points intraoperatively obtained. Moreover, the natural geometry of the trochlear groove likely scatters with the patient population [9, 18]. Similar to previous studies [3, 14, 34], a great variation in the trochlear groove’s geometric parameters were observed for the native femora and could explain the high standard deviations in our study. Consequently, the alterations we observed in SH are expected to cause a change in the patellofemoral joint kinematics and presumably higher patellofemoral pressure. These biomechanical parameters are indeed associated with anterior knee pain. Moreover, alteration of the SH might change the position of the patella and therefore the lever arm of the extensor muscles leading to differences in joint forces [9, 31]. Similar to the SH, our results showed distinct alterations in the parameters medCH and latCH due to TKA. In general, the femoral condyles, particularly the medCH, were higher in native knees. Varadarajan et al. [34] reported that the condylar height tended to be reduced after TKA. These findings are partly inconsistent with the results of the presented study. In our study, we found a tendency for the latCH to be increased due to TKA. On the opposite, the medCH was decreased after TKA which agrees with Varadarajan et al. [34] but again, the investigated implant designs were different between the studies and real TKA might be different from the simulation. In particular, the medial condyle was higher in the native knee compared to the prosthetic knees, which might lead to medial luxation postoperatively.

Lozano et al. [19] investigated the influence of mechanically and kinematically aligned total knee implants on the trochlear groove anatomy respective to the native knee. In agreement with our results, they found that the geometry of the trochlear groove was changed after TKA, e.g. the SH was changed by up to 5 mm while we detected a maximum and minimum mean difference by around 2 mm and −3.06 mm, respectively. The classification of all 33 patients showed that despite significant differences between preoperative and postoperative patellofemoral morphology, the majority received good reconstruction of the femoral geometry with a medium change (2.5–5 mm) in the investigated parameters. This is in accordance with the good clinical results of the investigated implant system [6].

Regarding the accuracy of the tactile scanner and its intraoperative use, the recorded points closely sampled the actual implant surface within a mean error in the distance of 0.79 mm, and an average RMSE of 0.65 mm. Thus, the raw data of our study can be considered sufficiently accurate. Depending on the number of recorded points and their spatial distribution, the generated surface matched the real geometry accurately; however, our presented approach represents some limitations. Despite a standardized procedure to sample the femoral surface, the data could not be recorded in the same way for each subject. Therefore, the quality of the reconstructed surfaces varied and suffered from interpolation artefacts and lack of coverage, especially in the outer regions. Hence, for very low (trochlear groove) and very high (anterior cortex) cutting angles θ, the automated parameter extraction was not always reliable. For the sake of quality and reliability, reconstructed surfaces were double checked for every patient. The reason behind all these limitations is the slow manual point acquisition procedure during surgery, which could lead to variations between different surgeons. This limitation represents the trade-off between experimental cadaver studies [14, 31], computational studies [7, 9, 34], and intraoperative data capture in living subjects. For future studies, our method will be improved as it concerns the sample point acquisition during surgery. In the present study, the trochlear bisector angle and sulcus position were not evaluated, although they are important parameters in knee implant design and should be included in future investigations. As we examined only one total knee replacement, the results are limited to this endoprosthesis design and it is desirable to extend the study not only to more parameters but also to a greater variety of implant designs. Another limitation is that no clinical data has been used, therefore, different morphological parameters may be correlated to the clinical outcome using standardised procedures and scores like the Knee Society Score or Oxford Knee Score in future studies [15]. Furthermore, the effect of implant positioning on the morphological key parameters of the patellofemoral joint should be analyzed in future studies as previously conducted by Lozano et al. [19].

Our study aimed to establish a method to intraoperatively investigate patellofemoral morphology before and after TKR. The approach enables intraoperative measurement of patellofemoral morphologies without radiation exposure. An inherent advantage of intraoperative analysis is the possibility to capture not only the implant design but also the actual implantation of the total knee endoprosthesis components depending on implant positioning and bone resection. In our feasibility study, a sufficient number of included patients could be realized to derive substantial findings [33] and the option to assess the postoperative situation of the trochlear groove [26] compared to the preoperative situation without radiation exposure. To our knowledge, only very few computational intraoperative investigations of patients before and after TKA exist, which highlights the importance of our present study.

In conclusion, the presented method proved to be capable of extracting important morphological parameters of the trochlear groove. Sulcus height and the height of the femoral condyles of the prosthetic trochlea differed from the native trochlea. Future work is needed to enable recruitment of a larger patient cohort and to include different implant designs, and clinical assessment of the correlation between morphological parameters and the postoperative outcome through clinical evaluation scores. This may support the further enhancement of surgical technique and design of femoral components for total knee replacements. Orthopedic surgeons should be aware of the possible changes in trochlear groove geometry. An increase in sulcus height will likely increase patellofemoral pressure and act as a contributor to anterior knee pain.

## Abbreviations

CAD: Computer-aided design
CR: Cruciate-retaining
CT: Computed tomography
latCH: Lateral femoral condyle height
medCH: Medial femoral condyle height
MRI: Magnetic resonance imaging
RMSE: Root mean square error
RP: Reference plane
SH: Sulcus height
TEA: Transepicondylar axis
TKA: Total knee arthroplasty
